# EuniceScope: Low-Cost Imaging Platform for Studying Microgravity Cell Biology

**DOI:** 10.1109/OJEMB.2023.3257991

**Published:** 2023-03-16

**Authors:** Wing Yan Chu, Kevin K. Tsia

**Affiliations:** University of Hong Kong 25809 Hong Kong; University of Toronto 7938 Toronto ON M5S Canada; Department of Electrical and Electronic Engineering, Faculty of EngineeringUniversity of Hong Kong 25809 Hong Kong

**Keywords:** Astrobiology, biomedical engineering, cell biology, cell imaging, microgravity, space health

## Abstract

Microgravity is proven to impact a wide range of human physiology, from stimulating stem cell differentiation to confounding cell health in bones, skeletal muscles, and blood cells. The research in this arena is progressively intensified by the increasing promises of human spaceflights. Considering the limited access to spaceflight, ground-based microgravity-simulating platforms have been indispensable for microgravity-biology research. However, they are generally complex, costly, hard to replicate and reconfigure - hampering the broad adoption of microgravity biology and astrobiology. To address these limitations, we developed a low-cost reconfigurable 3D-printed microscope coined EuniceScope to allow the democratization of astrobiology, especially for educational use. EuniceScope is a compact 2D clinostat system integrated with a modularized brightfield microscope, built upon 3D-printed toolbox. We demonstrated that this compact system offers plausible imaging quality and microgravity-simulating performance. Its high degree of reconfigurability thus holds great promise in the wide dissemination of microgravity-cell-biology research in the broader community, including Science, technology, engineering, and mathematics (STEM) educational and scientific community in the future.

## I. Introduction

SINCE the first human venture into space in 1961, space exploration has progressively been substantiated [1]. By 2020, 242 individuals from 19 countries have managed to travel to the International Space Station (ISS). Yet, the number of countries interested in the space sector is growing, as is the number of space agencies and the space economy. The success of new endeavors of human exploration of space and commercial spaceflights builds on understanding microgravity's physiological effects, which is critical to preserve astronauts' health in the prolonged space environment. A notable example is the study of muscle mass and strength loss, human tissue response to other stressors in space, among others [2].

The term ‘microgravity’ refers to the condition where the gravity appears to be very small [3]. The overall aim of this work is to demonstrate the feasibility of our low-cost system in accessing to this range of “microgravity” down to 10^−3^g. For the sake of simplicity, we refer to reduced gravity, hypo-gravity and microgravity as any value less than 1g in this paper.

Specifically, microgravity can induce cell morphological changes indicative of health and disease in astronauts under prolonged space exploration. For instance, normal human fibroblasts were found to grow as scaffold-free 3D multicellular spheroids rather than 2D monolayer growth on ground. In addition, spiculated erythrocytes (or red blood cells (RBCs)), a known marker of aging, were found more common under microgravity than spherocytic cells found on ground [4]. Different pathological conditions (e.g., anemia [5]) stem from impaired RBC, which are associated with RBC deformability [6] or surface morphological anomalies [4]. Besides, microgravity hinders cell growth behavior in musculoskeletal systems that could impact bone density and cartilage development [7], [8]. Weightlessness is also found to closely link to the tendency of stem cell differentiation towards different lineages [7].

Optical microscopy (imaging) has been instrumental in cell biology research for visualizing cellular characteristics from the organelle to the tissue level and cataloging human cell types, states, and functions. Notably, advances in optical imaging technologies have greatly catalyzed massive global initiatives (e.g., Human Cell Atlas, LifeTime Initiative in Europe) to craft high-resolution maps of cellular diversity of human biology and diseases [9], [10], [11]. Yet, real-time cellular microscopy/imaging for long-term space exposure study yet to be in the mainstream. A recent study reported the first real-time live cell imaging for observing mouse embryonic stem cell morphogenesis in space [12].

The resources for accessing outer space and, thus, the opportunities to launch space experiments on spacecraft are scarce. To circumvent this problem for enabling practical microgravity research, a handful of technologies offering ground-based microgravity simulation (GBS) has been established [13], [14], [15], including diamagnetic levitation [14], Rotating wall vessel (RWV) [16], Free falling machine (FFM) [17], 2D clinostat and random positioning machine (RPM) [15], [18], [19]. Among all, clinostat and RPM stand out for offering the solutions in terms of the practicality and robustness of microgravity generation (see Supplementary Table I). Clinostat rotates the sample along the horizontal axis while RPM involves two independent rotating frames around two axes [15], [18]. From the sample point of view, this rotational motion constantly re-orients the gravitational force (g-force) vector and effectively converges it toward zero in a time averaged sense. In essence, the samples experience negligible time-averaged g-force - simulating weightlessness [18], [19].

**TABLE I:** Summary of the Result of Image Quality Assessment

Resolution	Magnification	FOV	Aberration effects
0.69 μm	20X (20 times)	274.79 μm x 205.96 μm	Chromatic aberration

Although the technologies of clinostat/RPM are generally well-established, continuous real-time live cell imaging has not been widely adopted with the clinostat/RPM, except for a handful of demonstrations [20]. For instance, a dual-modality of digital holographic and fluorescence microscope was integrated with an RPM [21]. It operated in a time-sequential mode to study the real-time cytomorphological modifications in mouse myoblast cells [20]. Despite these successes, practical integration of optical microscopes with clinostat/RPM remains limited. The main challenge stems from the bulky footprint, sophisticated optics configuration and high cost of classical/advanced microscopes - making it difficult to adapt to clinostat/RPM operations without sacrificing imaging performance. This challenge arguably explains why the vast majority of the cellular behavioral imaging study under simulated microgravity have routinely been conducted a posteriori the fixation of cell samples, i.e., imaging is still performed separately from the clinostat/RPM operation. Real-time in-situ imaging under microgravity thus still currently incurs substantial instrumentation investment and development. Hence, space biology research has long been restricted to a handful of well-equipped labs and institutions and shied away from a broader biological research community. Here we attempt to question if this status quo can be overcome such that space biology research can be more easily disseminated and accessible with publicly available or easily accessible technology and tools, such as 3D printing, free online design software and opensource systems, which do not require special skills, termed “democratization”. Initial attempts to “democratizing” space biology research have been made. Examples include the development of desktop RPM, and integrating a handheld microscope with it [21]. Notably, this integration successfully visualized cell morphology and quantified the rate of cell cycle under simulated microgravity [22].

Motivated by an open 3D printed modular microscope development platform toolbox called UC2 (abbreviated as ‘YOU. SEE. TOO.’, i.e., users can experience how a microscope works or is constructed.) [23], we here introduce a low-cost and reconfigurable microscope, coined EuniceScope, integrated with a compact 2D clinostat system, all built by 3D printing. EuniceScope developed here demonstrated a new level of compactness, reconfigurability, and flexibility of a microscope tailored for microgravity cell biology. Its modularized nature allows easy modifications to be compatible with different microscopic modules and various types of samples. Compared with modern optical/microscope setups, which are immensely complex, the UC2 microscope system construction can easily be reconfigured according to different applications [24]. To the best of our knowledge, the adoption of UC2 for microgravity cell biology has yet to be demonstrated. We anticipate that this work could incentivize broader participation in space biology research and education and stimulate cost-effective technological developments in monitoring and investigating astronauts' health and disease in long run.

## II. Materials and Methods

### A. Bright-Field Microscope

#### 1) Design and Construction

The overall framework of the bright-field microscope was built upon the basic building block with a standardized cube size (50 mm on each side), which can accommodate the common optical components (e.g., mirrors and lenses). This standardized cubic configuration favors straightforward microscope assembly and optical alignment.

**Fig. 1.**
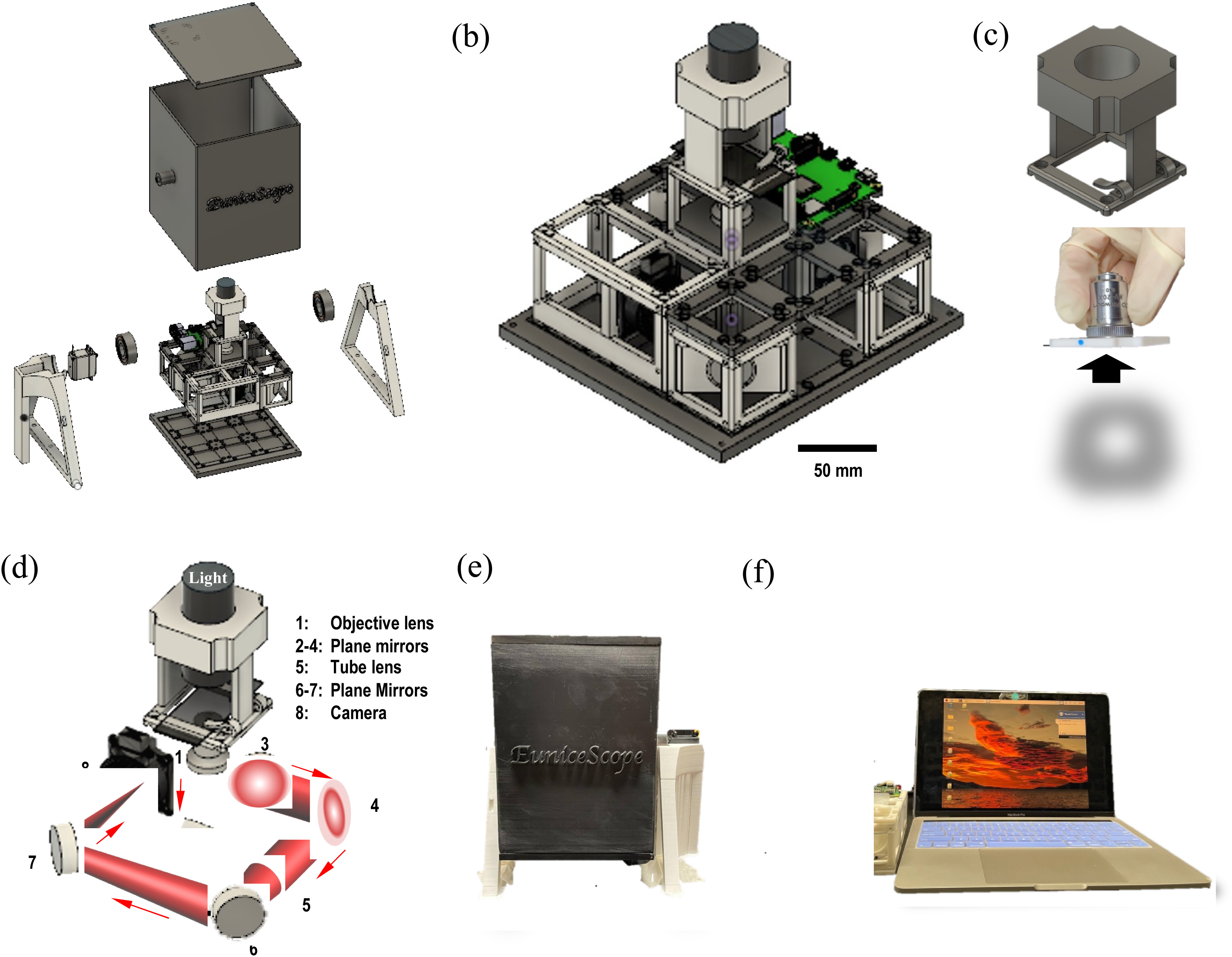
EuniceScope design and construction. (a) Clinostat design with microscope implemented (explosion view); (b) Microscope system (scale bar = 50 mm); (c) Sample stage with LED holder included (top), mounted objective (middle), objective mount (bottom); (d) 4f system configuration of the optical components (1-5: 200 mm; 5-8: 200 mm); (e) Constructed clinostat system; (f) Wireless control of the microscope system for image taking.

The bright field microscope adopts a standard infinity-corrected configuration (based upon a “*4f*” compound-lens system) with a magnification of 20X (see Fig. 1(a)–(c) and the details of this 4f configuration in Supplementary Figure 1). The key optical components include: a white-light source, a 20X objective (Newport MV-20X), 5 silver plane mirrors (Thorlabs), 1 infinity-corrected tube lens (Thorlabs). An infinity-corrected bright field microscope setup was chosen thanks to its advantages in reconfigurability, uniformity of sharp image focus across the field of view (FOV), improved contrast, and greater color clarity over the conventional finite corrected system [25] (see Supplementary Figure 2). Inserting additional optical elements to the “parallel optical path” region in the infinity-corrected microscope is possible without impacting the magnification. It thus allows flexible reconfiguration in the future (e.g., adding the polarizers to enable polarization light microscopy with the same system). To keep the system compact, the microscope architecture was designed in a double-deck configuration (see Fig. 1(a), (b) and (d)).

**Fig. 2.**
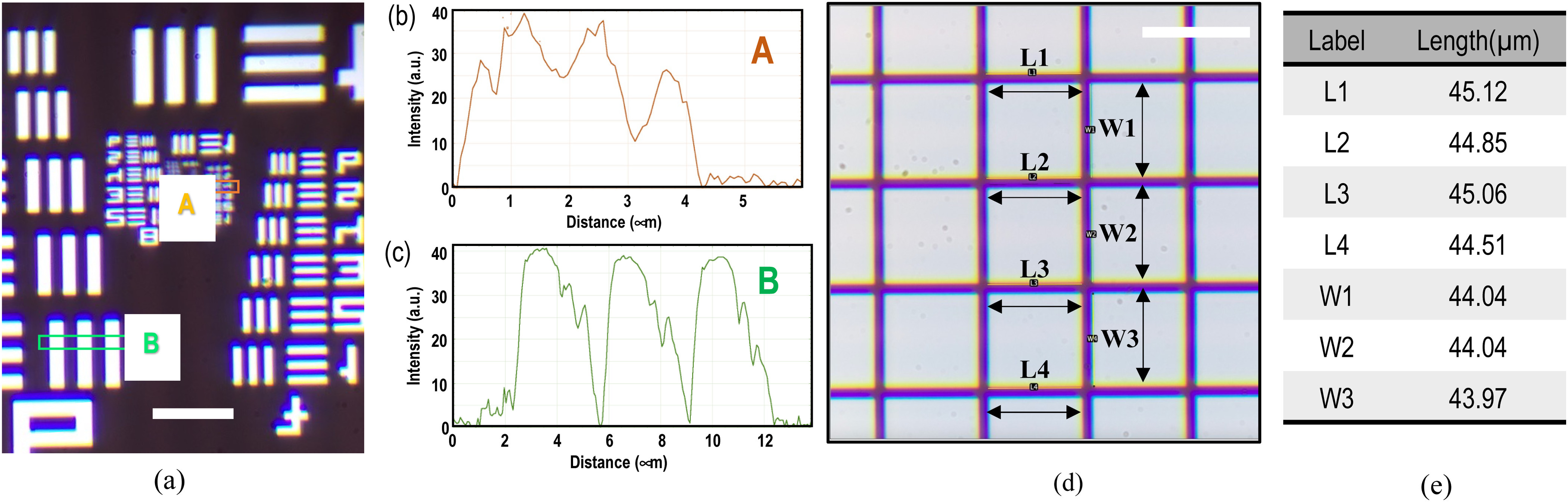
Image of resolution and distortion target with items selected for measurement. (a) Intensity profile of Group 9 Element 4 & Group 6 Element 1, the smallest visible Element 4 in Group 9 is pointed by red box, 2 elements are selected (red box A and green box B) for plotting intensity profile to show the resolving ability of the microscope system. The bar/space width calculation (microns) of this element is 0.691 μm.; (b) Intensity profile of area A; (c) Intensity profile of area B; (d) Image of distortion target with items selected for length measurement, in which their difference is insignificant, meaning no observed distortion (scale bar = 50 μm); (e) Length measurement of the selected items across the distortion target.

Several 3D-printed parts were custom-designed: an LED mount positioned above the sample stage, a UC2-compatible sample stage (Fig. 1(c)). To ensure robust and stable microscopy operation under continuous clinostat rotation, we also redesigned the original baseplate of the UC2 toolbox by introducing multiple studs on the building blocks. In this way, the building blocks and thus the optical components can be more tightly secured in place during rotation. In addition, we tailored an objective lens mount to be compatible this UC2-based microscope setup (Fig. 1(c)). A screw tap of Royal Microscopical Society thread drills 0.8-inch diameter × 36 thread-per-inch Whitworth thread form in the mount (Fig. 1(c)).

A built-in mini-computer Raspberry Pi 4B 4GB with HQ Camera (CSI-2 with 2 Megapixels Resolution) was positioned at the lower deck of the microscope for image capture. As the microscopic system was designed to operate inside a 2D clinostat, wireless control was adopted using a remote-control software (TeamViewer). This allows configuring the mini-computer and image recording with a remote computer via Wi-Fi (Fig. 1(f)). The remote-control tasks mainly include adjusting camera settings, recording raw images. The camera module is powered by a portable power bank (Verbatim: 10000mAh, 5V/3A output).

#### 2) Image Quality Assessment

The following image quality parameters were examined using a standard resolution target (Newport HIGHRES-2) and an open image processing/analysis tool (ImageJ/Fiji):

• Image resolution: The theoretic diffraction-limited resolution (D) is estimated as 0.69 μm based on Abbe Limit, which is expressed as
  \begin{equation*}\ \ \ D = \lambda /2NA \tag{1}
\end{equation*}

where $\lambda $ is the wavelength of the illumination light; $NA$ is the numerical aperture of the objective lens

• Image magnification
• Field of view area (FOV)
• Optical aberration effects

#### 3) Image Contrast

To improve image contrast, oblique illumination was adopted by adding a mask after the light source. Consequently, the details of an unstained (almost transparent) specimen could be revealed in a pseudo-3D manner (see Supplementary Figure 3).

**Fig. 3.**
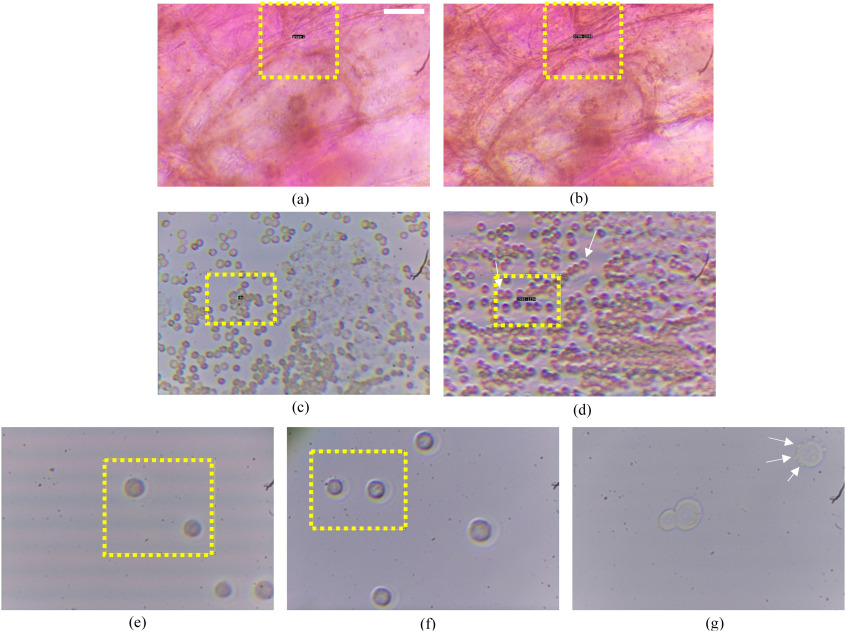
Image taken by the developed microscope system under full and oblique illumination (scale bar = 50 μm). Yellow boxes show the same area selected for intensity comparison under both illuminations in determining contrast for each sample. (a)-(b) Grape skin images; (c)-(d) RBCs; (e)-(g) H217 lung cancer cells; (a), (c) and (e) Full illumination; (b), (d) and (f) Oblique illumination; (g) Disintegration of H217 cell membrane under full illumination.

#### 4) Imaging Samples

To verify the functionality of microscopic system for cells/tissues imaging, various biological samples were tested. They include (i) plant cell imaging: grape skin (exocarp) and grape flesh (mesocarp); and (ii) animal cell imaging: human RBCs and lung cancer cells (H2170 cell line). Image focusing was done by manual adjustment of the sample stage, with other optical components fixed.

• *RBCs:* To prevent RBC aggregation that could obscure imaging quality assessment, 10 μl of RBCs was diluted to 0.9X concentration with 15 μl deionized water and 75 μl PBS-EDTA-BSA mixture. PBS was used to prevent osmotic cellular rupture. EDTA functioned as an anti-clotting agent of RBCs.
• *H2170:* The H2170 cells (from American Type Culture Collection (ATCC)) were cultured in the tissue culture flasks (surface area of 75cm^2^) (TPP), which was placed in a CO_2_ incubator with 5% CO_2_ under 37 °C. The full culture medium was ATCC modified RPMI-1640 (Gibco) supplemented with 10% fetal bovine serum (FBS) (Gibco) and 1% antibiotic–antimycotic (Gibco). Depending on cell confluency observed by a standard light microscope, passage or medium replacement was performed 2–3 times each week.

### B. Microgravity Simulation

A 2D clinostat was adopted over an RPM due to its simpler design and construction, robust mechanical stability and cost-effective performance in simulating microgravity based on biological samples (see Supplementary Table II).

#### 1) Hardware Design

Major hardware components include:

• Parallax feedback 360° high speed servo controlled by an Arduino Uno board was used for its sufficient torque of 2.2 kg-cm. It provides a wide range of rotational speed (0–140 rpm) for the study of angular frequency effect on microgravity simulation.
• Clinostat Box: The cover, body and bottom parts were designed to be UC2-compatible such that the brightfield microscope is enclosed and secured inside. For motor connection, a 25T-spline horn was redesigned for gluing onto the side of the box at a location similar to the sample level.
• 2 NPB 6204-ZZ Bearings (Mechatronics Bearing Group)
• 2 3D-printed supporting mechanical frames

#### 2) Rotational Speed Control

Rotational velocity of clinorotation is proven influencing microgravity simulation and cell behaviour [26]. An appropriate rotational velocity is needed to avoid cells from responding to gravity at low frequencies, and to limit the centrifugal force which introduces mechanical strains artifacts at high frequencies. Rotational speed of around 30–120 rpm is typically utilized for studying small animal samples in an aqueous growth medium [18], [27], [28]. Hence, 2 rotational velocities within this range were tested: 35 rpm and 90 rpm. A microcontroller (Arduino Uno) was employed and programmed to control the rotational speeds of motor.

#### 3) Clinostat Characterization

The peak centrifugal acceleration force driven by rotation is assumed to be the artificial gravity [19], [20] and the equation is:

\begin{equation*}
\alpha = {\omega }^2 \times r \tag{2}
\end{equation*}

where$\ \alpha $ [g] is the peak centrifugal acceleration force in unit Earth gravitational force; $\omega $ [rad/s] is the angular velocity; $r$ [m] is the distance of sample from the rotating axis. In this work, the calculated microgravity values are 0.0034 g at 35 rpm and 0.023 g at 90 rpm respectively.

A mobile phone with an accelerometer application (Physics Toolbox) was placed in the same position as the sample inside the clinostat system for 3 separate tests at different rotational speeds. The accelerometer measures the ratio of normal force to g-force in 3 dimensions: x, y, z directions. The collected data was analyzed and displayed using custom program (in Python). To verify the functionality of EuniceScope in cell imaging under microgravity simulation, images of plant cell (grape exocarp) was taken under rotation for every 30 seconds in 3 minutes.

## III. Results

### A. Bright-Field Microscopy

#### 1) Image Quality

An image of the resolution target was taken to reveal the following image quality: resolution, magnification, FOV and aberration effects (see Table I).

The captured intensity profiles showed that the lines (Group 9 Element 4), with the linewidth of 0.69 μm, can still be resolved. This is consistent with the predicted value according to the Abbe limit (see Fig. 2(b)). This proves that the setup achieves a submicrometer-to-micrometer imaging resolution (see also another intensity profile of Group 8 Element 1, in Fig. 2(c)).

From the distortion targets images, by measuring the length of the height and width of each grid box along the same column, there is neither obvious image distortion nor spherical aberration across the whole FOV (see Fig. 2(d) and (e)). Nevertheless, we observe mild chromatic aberration which is expected to confound our current experiments which do not involve the need for multi-color image assessment.

#### 2) Imaging Performance With Biological Samples

Imaging performance with the plant cells and animal cells using the designed microscope under full and oblique illumination are discussed as below. Quantitatively, compared to full field illumination, the image intensity contrast improvement by oblique illumination is 11% (at the cell wall for plant cells), 16% (RBCs) and 82% (H217) respectively (Table II). In general, the visual examination agrees with the calculated value. Notably, the image by oblique illumination also creates a pseudo-3D quality (Fig. 3(b), (d) and (f)).

**TABLE II:** Percentage Increase in Intensity in Different Samples

Sample	Illumination mode	Intensity (a.u.)			% Increase in Contrast
		Min	Max	Contrast (Max-Min)/(Max+Min)	
Grape Skin	Full	112	203	0.29	11%
	Oblique	104	203	0.32	
RBCs	Full	86	183	0.36	16%
	Oblique	87	212	0.42	
H2170	Full	106	175	0.25	82%
	Oblique	74	194	0.45	

The cell walls of the grape skin (exocarp) are generally distinctive with cell nuclei observable in exocarp images (see Fig. 3(a) and (b)), thanks to the 3D impression created by oblique illumination. On the other hand, the circular shape of RBCs is clearly captured with a biconcave disc shape revealed under oblique illumination (see Fig. 3(c) and (d)). Many shape changes in RBCs are pathologically associated, for example sickle-shape cells indicate anaemia etc. Hence, the ability to capture the shape and 3D impression under the developed microscope is significant. For cancer cell line H2170, cells morphology are clearly shown (see Fig. 3(e) and (f)). Cell membrane blebbing was clearly observed over time (see Fig. 3(g)).

### B. 2D Clinostat

The weight of the microgravity-simulating platform is about 2.5 kg with the dimensions of 264.8 mm × 263.8 mm × 206.7 mm. It is robust and stable to house and support the microscope system inside (Fig. 1(c) and (e)). Smooth rotation under different rotational speed was demonstrated.

**Fig. 4.**
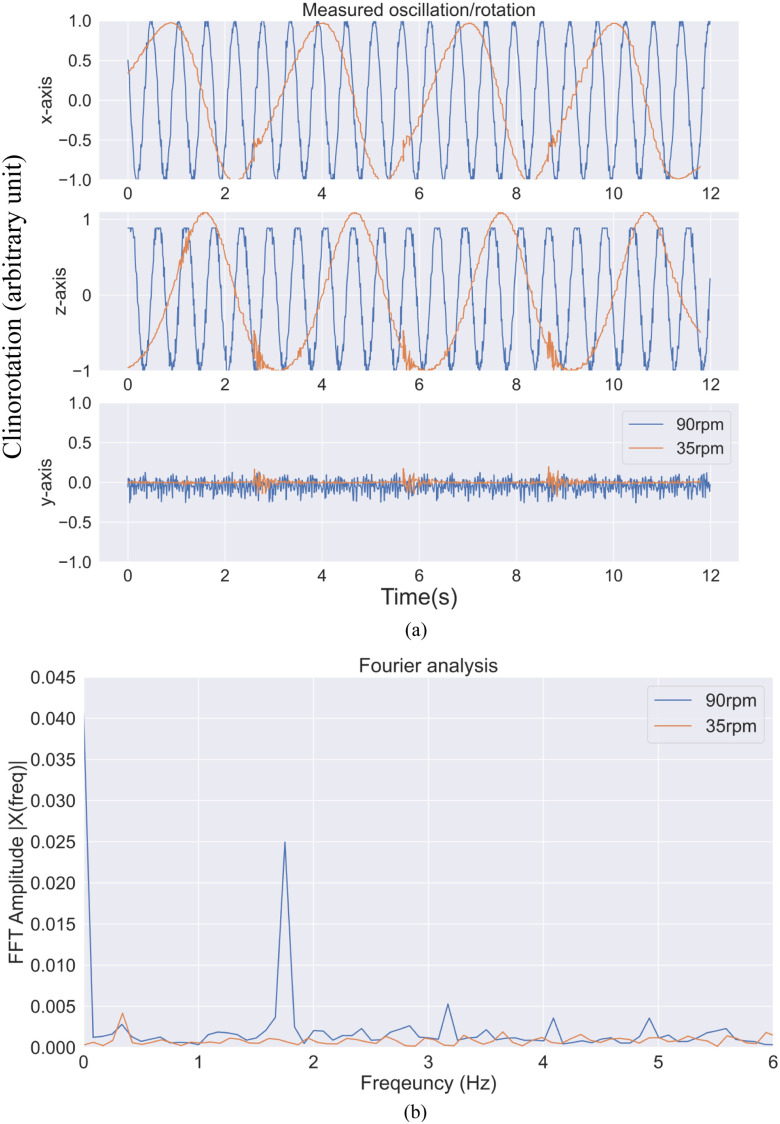
Plots for evaluating microgravity simulation quality. (a) G-force measurement at 35 rpm and 90 rpm (-ve: Opposite direction; GFx: G-force along x axis; GFy: G-force along y axis; GFz: G-force along z axis; TgF: Average g-force); (b) Fourier analysis for 35 rpm and 90 rpm rotation.

The g-force values measured along the x and z axes in both rotational speeds oscillate between −1g and 1g. The g-force along y-axis is generally stable at 35 rpm and 90 rpm, with minimal fluctuations (see Fig. 4(a)). From the Fourier analysis, the contributions of the higher harmonics of the oscillations in both cases (i.e., 35 rpm and 90 rpm) are measured to be insignificant - showing the rotation motion is highly sinusoidal (see Fig. 4(b)). The system is proved stable.

Using rotational speeds 35 rpm and 90 rpm, the simulated hypo-gravity values are in the order of 10^−2^ and 10^−3^ m/s^2^ respectively, in which the latter one satisfies microgravity definition. This shows the system is capable to achieve a simulation towards microgravity.

### C. Whole System (See Supplementary Table III)

**Fig. 5.**
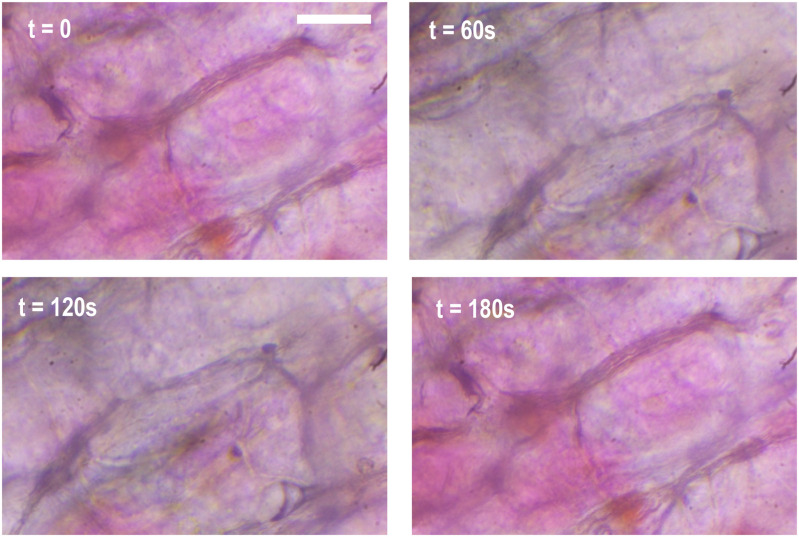
Images taken under rotation at 35 rpm for 3 mins (scale bar = 50 μm).

We observe slight sample drifting movement under rotation (see Fig. 5). This might be due to fluidic motion of the culturing medium and vibration as mentioned. However, the features (cell walls and subcellular textures) are shown clearly with distinguishable positions.

## IV. Discussion

### A. Bright-Field Microscopy

Typically, the size of plant cells and animal cells ranges from 1 μm to 100 μm. Hence, the high resolution of 0.69 μm and FOV of 275 × 206 μm is sufficient for resolving cells morphology. Besides, cell features and details are revealed in images, proving the potential in examining cell surface structural change under clinorotation.

With an adoption of oblique illumination, the edges and details of cells which are not visible under full illumination are shown with higher contrast. The increase in contrast by oblique illumination agrees with studies related to image quality assessment under oblique illumination [29], [30]. This result supports the theory that oblique illumination enhances image contrast both qualitatively and quantitatively [31], revealing more detailed and clear cell features. The observed chromatic aberration (see the explanation in Supplementary Figure 4) can be readily corrected by using the achromatic objective.

Overall, the bright-field microscope system achieved satisfying performance which is capable of capturing the structural change (cell membrane blebbing and disintegration) in cancer cell. This is significant as the capturing morphological change in cells under weightlessness is the ultimate goal of utilizing this microscope system.

### B. 2D Clinostat

A study compared the biological responses in GBS to that on space, mammalian samples showed similar response and that of other species had identical response [13]. Clinorotation method was found to induce biological response closer to real microgravity in space than diamagnetic levitation. Hence, using clinostat for this study is validated [13].

The 2D clinostat developed is lightweight and compact compared to established microgravity-simulating platforms, while robust enough for supporting the in-built microscope. Besides, the microscope system is able to take images in focus under rotation, proving its mechanical stability. In terms of the performance, the g-force verse time graph agrees with literatures [15]: a constant at 0 for the rotational axis (y-axis), and a changing sinusoidal curve between +1g and −1g in the other 2 axes (x and z axes) (see Fig. 4(a) and (c)). Phase shift between the 2 changing sinusoidal curve is also observed [15], proving the functionality of the system as expected.

Our generated values of gravity are in the order of 10^−2^ and 10^−3^ m/s^2^, which is within the range of reduced gravity for similar experimental applications [3], [7], [32], [33], [34], [35], [36], [37]. Slight deviations from the mean value were found driven by sources of error in the prototype design which we aim at improving in the next developmental stage.

First, there was an imbalanced weight along the rotational axis. The system weight is slightly more concentrated to the bottom due to the current microscope placement. It could result in a slightly uneven rotational speeds [38], which explains the discrepancies in the amplitudes around 0*g*. To counterbalance the weight, improvements has been made by rearranging the microscope components, including the peripheral devices, e.g., powerbank, Raspberry Pi board, and/or adding extra weight to the top of the system.

Second, although the simulated microgravity value at 90 rpm is smaller, oscillation is more vigorous observed in Fourier analysis. Possible causes include unbalanced load around the system's axis [39], [40], intensified by high rotational speed [41], wearing of the 3D-printed spline tooth on the detailed horn leading to loosened connection. This is one of the limitations of this system because an uneven rotation leads to a varying artificial gravity created at different time points, adding confounding factors to study the microgravity effect on cells. Wear-resistant metal spline tooth and gearless motor for lowering the noise and energy loss is suggested.

Another challenge is that 2D clinostat can only average one directional gravity rather than an actual microgravity, 2 influential axes left. The shifting weight distribution may lead to mechanical and bending stress, which has to be analysed accurately in examining effects on cells [18].

At this stage, although the microgravity value simulated is rather at the upper bound of ‘genuine microgravity’ experienced in space, it is already of importance as in to study other cellular behaviours in the reduced gravity scenario. Besides, for STEM education purposes, the simulated value is sufficient for demonstrating how microgravity could possibly be simulated and have an effect on biology, leaving rooms for inspiration and innovation.

Nonetheless, reaching microgravity in the order of 10^−6^ m/s^2^ is one of our ultimate goals, which we planned to achieve it by improving the robustness of current system, and adding an extra frame to an RPM, with 2 frames rotating with constant velocity and a random inversion of rotation direction, for better randomizing the gravity vector.

We note that clinostat is unable to perfectly reproduce the actual microgravity. Instead, it can average it by cancelling the directionality of the gravity vector. Yet, given their versatility and cost efficiency, it is an invaluable tool for many preliminary and exploratory research efforts. Further study requires more advanced system that could more practically simulate microgravity, e.g., 3D clinostat. We anticipate that our 3D-printed toolbox (i.e., EuniceScope) can readily be further upgraded to the designs of 3D clinostat, thanks to its highly modularized nature.

### C. Whole System

EuniceScope includes microscope and clinostat compartment made by publicly accessible software and technology with many online resources, holding a promising potential for future dissemination and even democratization.

Image quality by microscope and microgravity simulation by clinostat is satisfying. The operation is mechanically stable. From the images of cells taken under rotation, there are slight movement, possibly due to vibration effect as discussed and fluid motion surrounding the cells on the glass slide.

Besides, correct focus requires manual adjustment of the sample stage. The ideal position of sample is at the center of the rotational axis. Yet, due to the positional adjustment for focus, the radius of rotation is slightly varying, which might further introduce additional confounding factors to the quality of simulated microgravity. The manual adjustment of sample stage was a drawback to the experiment operational efficiency, so an implementation of motorized sample stage will be explored in the next model.

Microgravity measurement using mobile phone applications also possibly introduce measurement error because the position of inbuilt accelerometer is unknown, and the phone might be positioned differently for each measurement due to manual operation for this part. Nonetheless, the use of accelerometer app in the first step serves as a proof of concept that microgravity measurement is possible with an accelerometer. Hence, an integrated accelerometer is suggested for a controlled measurement in the next step.

For the sample-holding method, microfluidic channels (PDMS-coated glass slide) is also suggested to reduce the fluid's freedom of movement. Nevertheless, the images are of good contrast with capabilities of capturing cell morphological changes. Sample holding method has also to be able to keep cells alive under microgravity within the whole image-taking duration. Implementing an in-built portable miniaturized low-cost long-term live-cell imaging platform into the system is optimal and proved promising [42]. A design for a rotatable incubator with size 75 cm × 70 cm × 56 cm is proposed (see Supplementary Figure 5).

## V. Conclusion

EuniceScope developed in this project achieved plausible quality in imaging and microgravity simulation. It also displays compactness, reconfigurability and flexibility which are the advantages over current systems. Democratizing capabilities are demonstrated with the use of opensource toolbox UC2, 3D printing technology, easily accessible software (e.g., Fusion 360) and electronic components (Raspberry Pi). These advantages allow on-ground space cell biology studies and promotional astrobiology in STEM education. Improvements on image quality and microgravity simulating stability are identified. Further development includes an in-built incubator that favors practical biological study under simulated microgravity. This system holds a high potential for assessing health impact of microgravity in a wider community prior popularization of space travel, ensuring health and safety of astronauts and potential space travelers.

## Supplementary Materials

Supplementary materials
