# Supplementary material for: EuniceScope: Low-Cost Imaging Platform for Studying Microgravity Cell Biology
Source: IEEE Open J Eng Med Biol. 2023 Mar 16;4:204–11. doi: 10.1109/OJEMB.2023.3257991 (PMC10810312; doi:10.1109/OJEMB.2023.3257991)
Supplement: Supplementary materials [file supp1-3257991.pdf]

Supplementary Materials

EuniceScope: Low-cost imaging platform for studying microgravity cell biology

Wing Yan Chu and Kevin K. Tsia

Table I. Working principles and properties of 5 common microgravity simulating systems

	**2D clinostat**	**RPM**	**RWV**	**Diamagnetic levitation**	**FFM**
**Working principle [1, 2]**	Rotation along 1 axis	2 frames with randomized movement	Constant rotation to prevent cells from settling	A high gradient magnetic field prevents sedimentation	Free fall for 800 ms, ‘bounce’ of 20g for 50 ms
**Microgravity duration [3]**	Hours to weeks			Minutes to hours	2.2s – 9.5 s
**Microgravity quality [3]**	$\leq 10^{-3}$ g	10^{-4} g	$\leq 10^{-3}$ g	$<10^{-2}$ g	10^{-6} g
**Application [3]**	1. Cells<br>2. Microbes<br>3. Plants			1. Cells<br>2. Microbes<br>3. Plants<br>4. Animals	1. Cells<br>2. Microbes<br>3. Plants
**Limitations when compared to 2D clinostat and RPM**			1. Cells are immersed in vessels or cylinders,<br>2. Difficult to implement microscopic systems	1. Space-occupying<br>2. High standards components (limited access)<br>3. Undesirable for democratization	1. Short period of microgravity effects<br>2. Require many periodic cycles<br>3. Not applicable for long term microgravity simulation studies

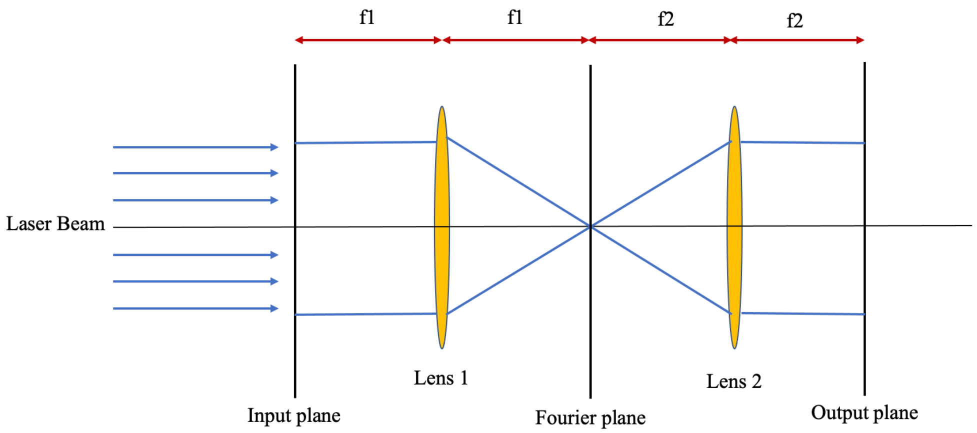

Fig. 1 4f configuration mechanism. The 4f system is an optical relay consists of two positive lenses with the input plane located one focal length (f1) in front of Lens 1 and the output plane located one focal length (f2) after Lens 2, and the magnification is −f2⁄f1.

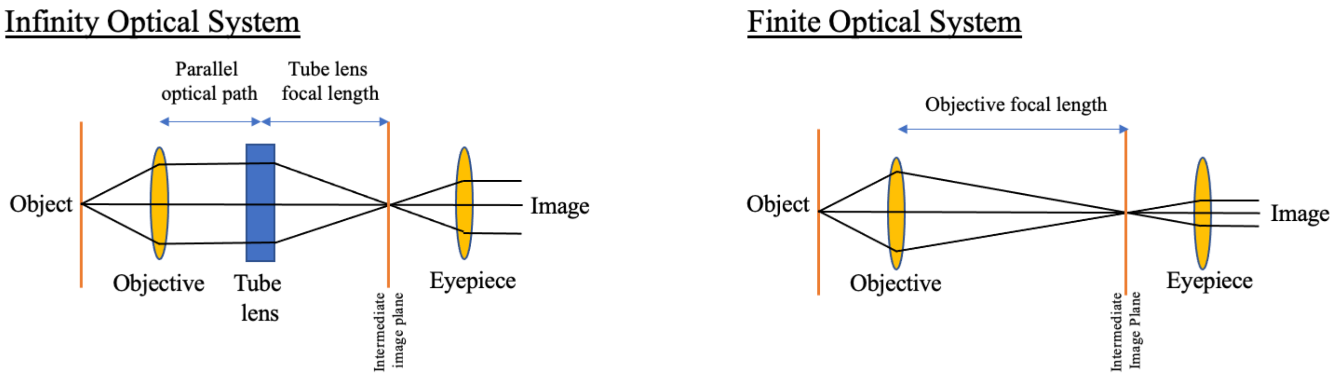

Fig. 2 Mechanism of infinity-corrected optical system (left) and the comparison with finite optical system (right). A parallel light beam instead of converging light beam is passed between the objective lens and tube lens. Therefore, despite a changed distance between objective lens and tube lens, the magnification is not changing [4]. Addition of auxiliary optical elements between the objective lens and tube lens is allowed with an unchanged constant parfocal point, no image shift is resulted [5]. This allows convienient modifications for different applications.

Table II Detailed comparison of 2D clinostat and RPM

	**2D clinostat**	**RPM**
**Components**	1 horizontally rotating frame	2 frames perpendicularly positioned
**Rotation mechanism [1, 6]**	1 axis of rotation:<br>weightlessness along its axis of rotation	2 frames rotate separately in several operational modes:<br>average gravitational pull over all directions to 0
**Force calculation [1]**	a = ~ 0.08g	a = ~ 0.05g
**Performance [1]**	• Induces less vibration<br>• Reveal comparable results as in real microgravity	• Induces further vibrations<br>• Not suitable in its random mode for macrophage studies<br>• No further advantages over clinostat

Table III Overall specifications of EuniceScope

**Composition**	**Weight**	**Dimension**
1. Brightfield Microscope<br>2. 2D clinostat	0.82 kg	264.8 mm x 263.8 mm x 206.7 mm

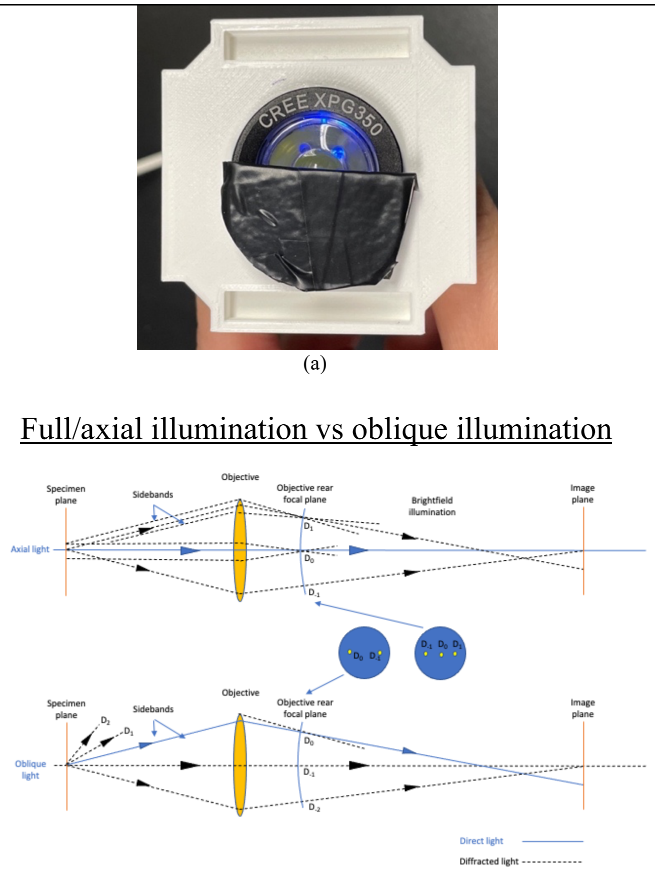

(a)

(b)

Mechanism:
Oblique illumination is also known as anaxial illumination. By adding a mask to the light source, it restricts the directed light from light source to a single azimuth, resulting in a single-sided illumination to the specimen. Thus, the zeroth order of light is displaced from the center to one peripheral side of the objective aperture. This allows an inclusion of additional higher orders of diffracted light at the objective rear focal plane [7]. Constructive and destructive interference is resulted between the undeviated light and the remaining sideband. Due to this uneven illumination and an effective doubling of angular aperture, an contrast-and-resolution-increased image is formed at the intermediate image plane.

Under full illumination, the diffracted light by the transparent specimen is ¼ wavelength out of phase with respect to the undeviated light at the image plane. This causes a lack of significant contrast and poor visibility, which explains the resulting image of unmasked light source in this project.

Fig. 3 Oblique illumination implementation and mechanism. (a) Masked LED; (b) Oblique illumination mechanism [7].

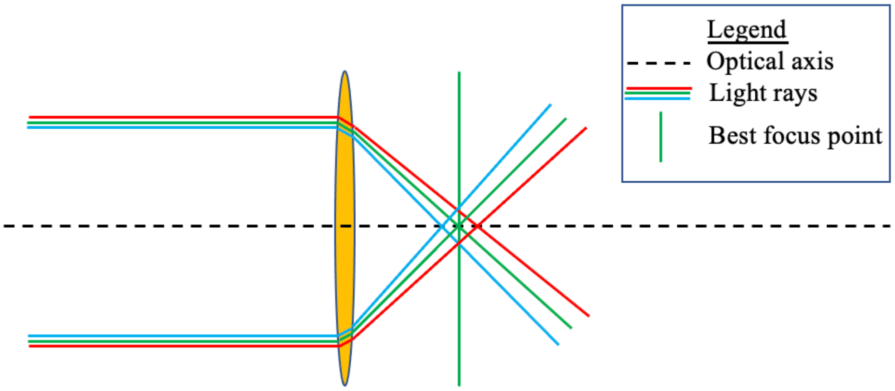

Fig. 4 Chromatic aberration. As white light from the LED headlight passes through the lens system, the component wavelengths refracts with respect to their frequency. Typically, the refractive index of glass is greater for shorter (blue) wavelengths and changes rapidly with a decreasing wavelength. Hence, chromatic aberration is observed.

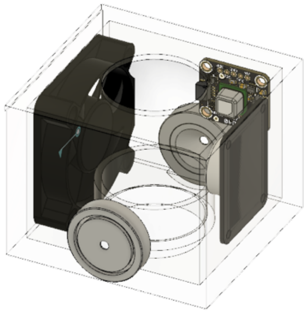

Fig. 5 Proposed design of rotatable incubator. The suggested casing material is glass or transparent polymer. Ball bearing assisted rotation implementation (grey parts) is suggested, the central hole allows penetration of gas pipes and wires. To regulate the temperature in the incubator environment, one sensor with the ability to sense CO2, humidity and temperature; and one O2 sensor are suggested, reducing the sensors included for compactness. Further fabrication and testing is needed to validate the functions of the proposed design of rotatable incubator.

REFERENCES

[1] S. Brungs, J. Hauslage, and R. Hemmersbach, "Validation of Random Positioning Versus Clinorotation Using a Macrophage Model System," *Microgravity Science and Technology,* vol. 31, no. 2, pp. 223-230, 2019/04/01 2019, doi: 10.1007/s12217-019-9687-0.

[2] S. Brungs *et al.*, "Facilities for Simulation of Microgravity in the ESA Ground-Based Facility Programme," (in English), *Microgravity Science and Technology,* vol. 28, no. 3, pp. 191-203, Jun 2016
2016-06-22 2016, doi: http://dx.doi.org/10.1007/s12217-015-9471-8.

[3] F. Ferranti, M. Del Bianco, and C. Pacelli, "Advantages and Limitations of Current Microgravity Platforms for Space Biology Research," *Applied Sciences,* vol. 11, no. 1, p. 68, 2021. [Online]. Available: https://www.mdpi.com/2076-3417/11/1/68.

[4] olympus-lifescience. "Infinity-corrected Optical System." https://www.olympus-ims.com/en/microscope/terms/feature15/#:~:text=Advantages%20of%20Infinity%2Dcorrected%20Optical,and%20tube%20lens%20is%20changed (accessed 2022, April 9.

[5] Scot Ellis, "Advantages of Infinity-Corrected Optics in
FT-IR Microspectroscopy." [Online]. Available: https://tools.thermofisher.com/content/sfs/brochures/D10261~.pdf

[6] C. Ulbrich *et al.*, "The impact of simulated and real microgravity on bone cells and mesenchymal stem cells," (in eng), *Biomed Res Int,* vol. 2014, p. 928507, 2014, doi: 10.1155/2014/928507.

[7] olympus-lifescience. "Oblique Illumination." https://www.olympus-lifescience.com/en/microscope-resource/primer/techniques/oblique/obliqueintro/ (accessed 2022, April 12).
